# Vaccine-related misinformation and attitude toward COVID-19 vaccination in Japan

**DOI:** 10.3389/fpubh.2026.1805589

**Published:** 2026-07-31

**Authors:** Yuki Furuse, Takahiro Tabuchi

**Affiliations:** 1 Department of Microbiology and Infection, The University of Tokyo Pandemic Preparedness, Infection and Advanced Research Center (UTOPIA), The University of Tokyo, Tokyo, Japan; 2 Department of Medical Virology, Nagasaki University Graduate School of Biomedical Sciences, Nagasaki, Japan; 3 Division of Epidemiology, School of Public Health, Tohoku University Graduate School of Medicine, Sendai, Japan; 4 Cancer Control Center, International Cancer Institute, Osaka, Osaka, Japan

**Keywords:** COVID-19, misinformation, online survey, vaccination, vaccine hesitancy

## Abstract

**Background:**

Misinformation about vaccines has been linked to vaccine hesitancy, but vaccination can still be accepted even when such distrustful narratives are endorsed. Evidence on how often this occurs and which factors shape vaccine acceptance among individuals who endorsed misinformation remains limited.

**Methods:**

We analyzed data from the Japan COVID-19 and Society Internet Survey conducted in September–October 2021 among 31,000 panel participants aged ≥15 years. Participants were asked about their COVID-19 vaccination status or attitudes, and about their agreement with seven vaccine-related misinformation statements. We used inverse probability weighting to calculate prevalence and proportions of answers, adjusting for population stratification in Japan. We also estimated odds ratios for factors associated with vaccination intention, stratified by misinformation endorsement status.

**Results:**

Among respondents who had been vaccinated, 8.1% endorsed at least one misinformation statement, whereas 36.6% of vaccine-hesitant individuals did so. Among individuals who endorsed at least one misinformation statement, 63.6% still accepted vaccination. In stratified multivariable models, older age was strongly associated with vaccine acceptance among individuals who did not endorse misinformation statements, whereas among individuals who endorsed misinformation, younger adults showed higher acceptance. The effect of information from governments on vaccine acceptance was significant only among individuals who did not endorse misinformation. The positive association of television/newspaper and the negative effect of internet/social media on vaccine acceptance were stronger among misinformation endorsers than non-endorsers.

**Conclusion:**

Endorsement of vaccine-related misinformation statements was associated with lower vaccine acceptance, yet a majority of misinformation endorsers still accepted vaccination. Determinants of vaccine acceptance differed by endorsement status, particularly by age and information sources, underscoring the need for nuanced communication approaches that address both misinformation narratives and broader trust dynamics.

## Introduction

The COVID-19 pandemic has caused substantial morbidity and mortality worldwide since its emergence. To mitigate its impact, COVID-19 vaccines were developed and deployed at unprecedented speed (1). Vaccination has long been one of the most effective public health measures for preventing severe illness and reducing hospitalizations, as illustrated by the impact of established programs such as pneumococcal and rotavirus vaccination (2–4). During the COVID-19 pandemic, access to routine, non-COVID-19 health services was also substantially disrupted in Japan and other countries due to diverted healthcare resources, limited supplies, and many patients avoiding care (5–7). Maintaining high vaccine uptake was important not only for prevention of infection and severe illness, but also for helping preserve healthcare system capacity during a period of exceptional strain (8, 9).

In Japan, COVID-19 vaccination began in February 2021, initially prioritizing healthcare workers and people at higher risk of severe illness, followed by rollout to the general population. Vaccine coverage in the country increased rapidly and reached approximately 80% by the end of 2021 (10).

Despite the high overall coverage, vaccine hesitancy persisted in a subset of the population (11, 12). Prior studies have identified sociodemographic factors (e.g., younger age, lower income, and lower educational attainment) and information environments as associated with vaccine hesitancy in Japan and elsewhere (11, 13–16).

During the pandemic, misinformation and conspiratorial narratives related to COVID-19 and vaccines also spread widely (17, 18). These narratives included allegations of data fabrication or concealment by governments or pharmaceutical companies (19) and clinical-biological claims about vaccine harms, such as infertility, genetic alteration, and the implantation of microchips (20, 21). Such misinformation has been repeatedly identified as an important contributor to vaccine hesitancy and distrust. A growing literature links exposure to and endorsement of such misinformation to vaccine hesitancy (22–25).

However, vaccination can still be accepted among individuals who endorse such distrustful narratives. The coexistence of misinformation endorsement and vaccine acceptance suggests that endorsement does not deterministically translate into behavior, and that determinants of acceptance may differ across endorsement strata. This has practical implications for communication strategies that seek to increase vaccination uptake during ongoing and future public health crises (26, 27).

In particular, media exposure has been increasingly recognized as a key determinant of vaccine attitudes and behavioral intentions. Studies have suggested that both traditional and digital media can shape vaccine confidence, hesitancy, and uptake, with effects that vary according to platform, content, and information quality (28–31). This complexity provides an important rationale for examining how different information sources relate to vaccine acceptance in the context of widespread misinformation.

In this study, using the data of the Japan COVID-19 and Society Internet Survey (JACSIS), we aimed to quantify the proportions of vaccine acceptance and hesitancy among individuals who did and did not endorse misinformation. We further explored factors, such as information sources, associated with misinformation endorsement and examined whether factors associated with vaccine acceptance differed by misinformation endorsement status. Clarifying why some individuals still accept vaccination despite endorsing misinformation is important from a public health perspective, because it helps sustain vaccine uptake even among individuals who hold false or distrustful beliefs. Such knowledge can inform communication and intervention strategies not only to counter misinformation itself, but also to preserve or improve vaccine acceptance during the era of infodemics.

## Materials and methods

### Study design and participants

This cross-sectional study analyzed data from the JACSIS, a nationwide internet-based survey administered by a commercial survey company, Rakuten Insight, with a large national panel of 2.2 million individuals, conducted annually since 2020 (32, 33). The results of the second wave of JACSIS were analyzed in the present study, which enrolled 31,000 participants aged 15 years or older between September and October 2021.

A target sample size was determined in the first wave of the JACSIS study in 2020 to secure adequate numbers across sex and age strata to support population-adjusted analyses (33–35). Eligible panel members were invited until quotas were met. To improve generalizability in calculating the prevalence and proportions of each answer, we applied inverse probability weighting based on national survey data, the Comprehensive Survey of Living Conditions in 2019 (36), using pre-specified covariates including residential prefecture, education level, marital status, and self-rated health consciousness (35, 37). Still, it should be noted that, because participants were recruited from an internet panel, individuals with limited internet access or low digital literacy may have been underrepresented in the survey.

### Measures

COVID-19 vaccination status and attitudes were assessed using a six-category item: fully vaccinated, partially vaccinated, willing but unable due to medical reasons, willing but not yet vaccinated (including those who were booked or seeking an appointment), undecided, and unwilling. For primary analyses, we excluded respondents who were unable to be vaccinated for medical reasons. We defined vaccine acceptance as fully/partially vaccinated or as willing but not yet vaccinated, and defined vaccine hesitancy as undecided or unwilling. Because almost all Japanese citizens aged 12 or older had been eligible for vaccination and offered the opportunity to receive it by the time of the survey, “undecided” was classified as hesitancy in this study. To assess the consistency of this grouping, we performed a sensitivity analysis as described later.

Endorsement of vaccine-related misinformation was measured by agreement with seven statements concerning alleged vaccine data fabrication or concealment (Table 1). The seven statements were adopted from a study that had validated them to measure vaccine conspiracy beliefs on human papillomavirus vaccine acceptance (19). Responses were recorded on a seven-point Likert scale ranging from “strongly disagree” to “strongly agree”; endorsement was defined as selecting “agree” or “strongly agree.”

**Table 1:** Seven vaccine-related misinformation statements.

Misinformation statements asked in the JACSIS study
Vaccine safety data is often fabricated.
Immunizing children is harmful and this fact is covered up.
Pharmaceutical companies cover up the dangers of vaccines.
People are deceived about vaccine efficacy.
Vaccine efficacy data is often fabricated.
People are deceived about vaccine safety.
The government is trying to cover up the link between vaccines and autism.

The statements were adopted from https://doi.org/10.1016/j.pvr.2016.09.001.

Explanatory variables were selected based on prior knowledge from previous studies and a systematic review (11, 13–16, 23, 27, 38, 39) and included age, sex, educational attainment, household income, physical comorbidity, mental illness, infection history of COVID-19, and information sources regarding COVID-19 and vaccination (family, friends, medical doctors, experts, government, internet/social media, newspapers, and television).

### Statistical analysis

We investigated the prevalence of misinformation endorsement by vaccination attitude (acceptance vs. hesitancy) and examined the proportions of vaccine acceptance and hesitancy by endorsement status. Factors associated with vaccine hesitancy were assessed using a binomial logistic regression model. In this analysis, the hesitancy group included both “unwilling” and “undecided” individuals. As a sensitivity analysis, we also conducted a multinomial logistic regression in which the “unwilling” and “undecided” subgroups were analyzed separately. Factors associated with the number of endorsed statements were investigated using an ordered logistic regression model.

To examine whether associations of possible risk factors with vaccination attitude differ by endorsement status, we fit logistic regression models separately, stratifying by endorsement status: one group of individuals who endorsed two or more statements, and another group who did not endorse any misinformation statements. Odds ratios (OR) adjusted for all variables, including age, sex, educational attainment, household income, physical comorbidity, mental illness, infection history of COVID-19, and information sources, tested with 95% confidence intervals (CI), were calculated. Because we used a single multivariable logistic regression model to estimate adjusted odds ratios of multiple factors simultaneously, standard multiple comparison corrections, such as Bonferroni correction or Benjamini-Hochberg procedure for multiple tests, are not required. Statistical significance was defined as two-sided *p* < 0.05. All analyses were performed in R (version 4.3.2).

### Ethics

The JACSIS study protocol was reviewed and approved by the Institutional Review Board of the Osaka International Cancer Institute (No. 20084). Online informed consent was obtained from participants. Data were anonymized prior to analysis.

## Results

Of 31,000 participants, 28,175 (90.9%) provided valid responses. Respondents aged between 15 and 80; the sample size for the age group of 15–19 years was 573, and each of the other 10-year age groups consisted of >3,500 individuals (Supplementary Table S1). At the time of the survey, COVID-19 vaccines were widely available to eligible age groups in Japan. Among valid responders, 83.2% (*n* = 23,435) reported being already vaccinated, 3.4% (968) were willing but not yet vaccinated, 6.2% (1,740) were undecided, 6.4% (1,800) were unwilling, and 0.8% (232) were unable to be vaccinated for medical reasons.

Overall, 88.9% (25,041) of participants endorsed none of the seven vaccine-related misinformation statements (Supplementary Table S1). We then adjusted our data to the general population of Japan using inverse probability weighting to calculate prevalence and proportions, taking into account residential prefecture, education level, marital status, and self-rated health consciousness.

**Figure 1:**
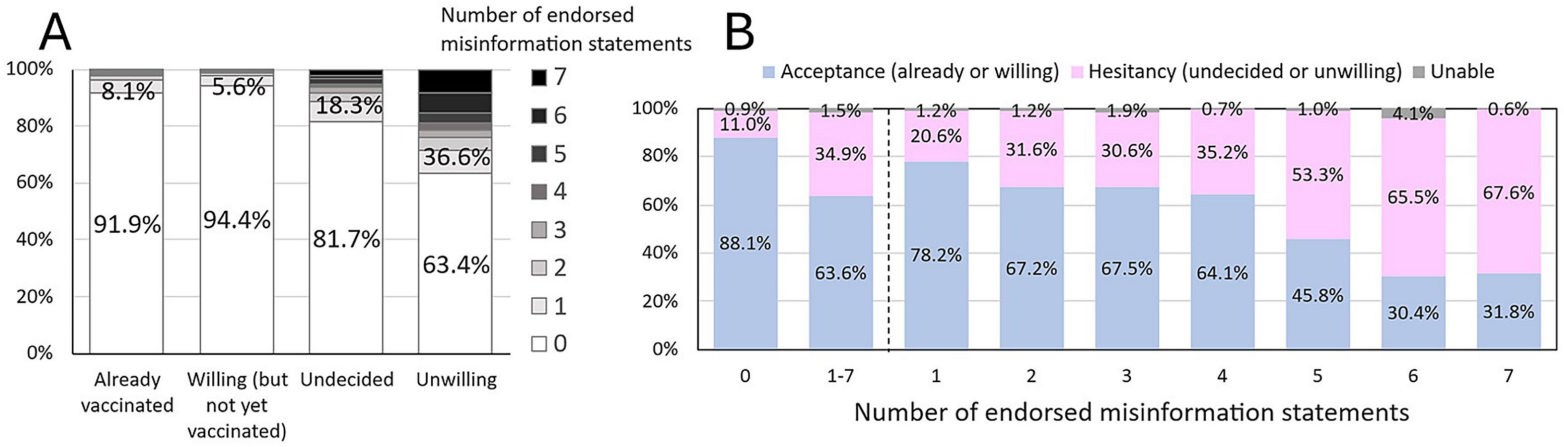
Prevalence of vaccine hesitancy and misinformation endorsement. **(A)** The prevalence of individuals who endorsed vaccine-related misinformation statements was stratified by vaccination status and attitude. The percentage of zero misinformation statement endorsement and the total percentage of 1–7 misinformation statements endorsement are labeled. **(B)** The proportions of vaccine acceptance and hesitancy were shown, stratified by the number of endorsed misinformation statements. “1–7” indicates people who endorsed at least one of the seven vaccine-related misinformation statements. The survey results were adjusted for age, residence, education, marital status, and self-rated health consciousness to fit the general population in Japan. The results for each age group are shown in Supplementary Figure S1.

Among those already vaccinated, 8.1% endorsed at least one misinformation statement, whereas 36.6% of those unwilling to be vaccinated endorsed at least one statement (Figure 1A). Viewed from the opposite direction, vaccine acceptance still remained common among individuals who endorsed such misinformation statements; 63.6% of misinformation endorsers were vaccinated or willing to be vaccinated (Figure 1B). Vaccine acceptance declined as the number of endorsed misinformation statements increased.

In multivariable models, endorsement of vaccine-related misinformation statements and vaccine hesitancy shared several associated risk factors, including younger age, lower income, and reliance on the internet/social media for COVID-19 information (Supplementary Tables S2, S3). The hesitancy group in this analysis included both “unwilling” and “undecided” individuals. As a sensitivity analysis, we separated the hesitancy group into “unwilling” and “undecided” subgroups and found that the risk factors identified above remained statistically significant in both subgroups (Supplementary Table S4). In those statistical tests, demographic and socioeconomic factors, including age, sex, educational attainment, household income, physical comorbidity, mental illness, COVID-19 infection history, and information sources, were included in the adjustment. Still, endorsement of misinformation statements was strongly associated with vaccine hesitancy (adjusted OR of 2.12 [95% CI 1.77–2.52] for one misinformation statement endorsement and 14.99 [95% CI 11.34–19.93] for seven statements).

**Figure 2:**
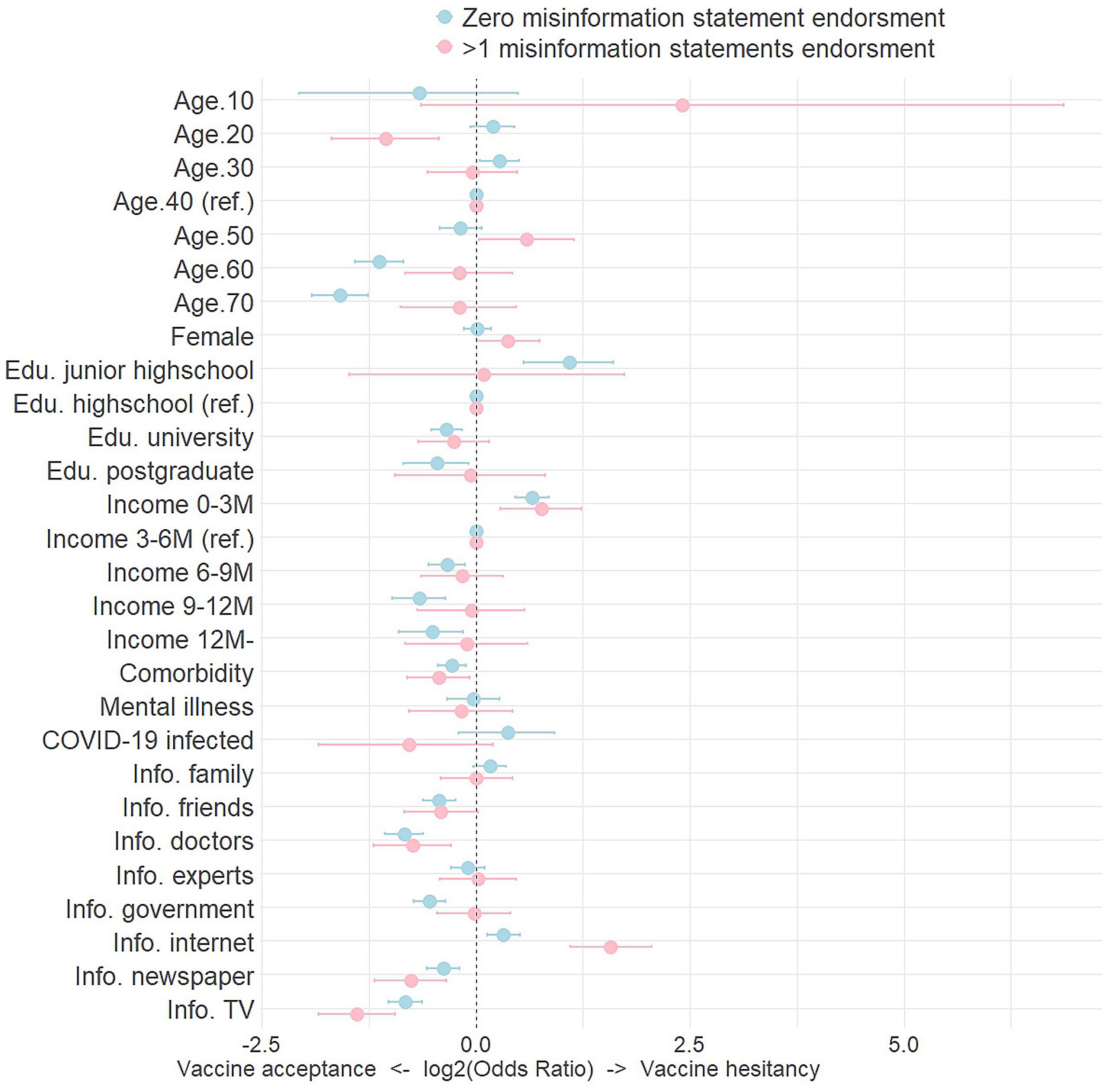
Factors associated with vaccine hesitancy in individuals who did and did not endorse misinformation. The adjusted odds ratios for vaccine hesitancy in individuals who endorsed none of the seven vaccine-related misinformation statements (blue) and those who endorsed two or more misinformation statements (pink) are shown. Statistical adjustment was performed using a multivariable model that included all variables shown in the figure. Horizontal lines indicate 95% confidence intervals. Age., age group; Edu., educational attainment; Info., Information source; ref., reference. Income is in million Japanese Yen. Data values are provided in Supplementary Table S5.

We then examined associations between vaccine hesitancy and risk factors, stratified by misinformation endorsement status (Figure 2; Supplementary Table S5). Many associations were consistent across strata. For example, obtaining information from medical doctors was associated with vaccine acceptance in both groups, whereas lower income was commonly associated with vaccine hesitancy.

Notable differences were observed for age and some information sources. Among individuals who endorsed none of the misinformation statements, older age showed significantly lower odds ratios for vaccine hesitancy (e.g., age 60–69 vs. 40–49: adjusted OR 0.46 [95% CI 0.38–0.56]), while there was no significant difference between age 20–29 vs. 40–49 (adjusted OR 1.15 [95% CI 0.96–1.37]). However, among misinformation endorsers, younger adults, in turn, showed a negative association with vaccine hesitancy (age 20–29 vs. 40–49: adjusted OR 0.48 [95% CI 0.31–0.74]), whereas older age was not consistently associated with lower hesitancy. Because only 49 individuals in the 15–19-year age group endorsed more than one misinformation statement (Supplementary Table S1), the corresponding estimate for the age group was statistically imprecise and should be interpreted with caution, as indicated by the wide confidence interval.

Additionally, information from governments was significantly associated with lower vaccine hesitancy only among individuals who did not endorse any misinformation statement (adjusted OR 0.69 [95% CI 0.61–0.78]), whereas it was not among misinformation endorsers (adjusted OR 0.98 [95% CI 0.73–1.32]). Acquiring information from newspapers and television showed a negative association with vaccine hesitancy. The effect was stronger among misinformation endorsers than non-endorsers (Figure 2). For example, regarding television, the adjusted OR was 0.38 [95% CI 0.28–0.52] for misinformation endorsers and 0.56 [95% CI 0.49–0.65] for non-endorsers. Similarly, the positive association between internet/social media information and vaccine hesitancy was stronger among misinformation endorsers (adjusted OR 2.97 [95% CI 2.14–4.14]) than among non-endorsers (adjusted OR 1.25 [95% CI 1.10–1.43]).

## Discussion

In this nationwide internet survey conducted in Japan, we found that factors such as age, income, and information sources are strongly associated with both endorsement of vaccine-related distrustful narratives and vaccine hesitancy, aligning with international studies linking the spread of misinformation and low vaccination intention (11, 22–25, 39, 40). We extend this work by quantifying the extent of vaccine acceptance among misinformation endorsers and by examining factors associated with vaccine acceptance within misinformation endorsement strata.

We found that a majority of misinformation endorsers still accepted vaccination, although endorsement of misinformation was strongly associated with lower vaccine acceptance. The finding highlights that endorsement of misinformation and behavior do not map one-to-one. Furthermore, we showed that determinants of vaccine acceptance and hesitancy may differ between individuals who did and did not endorse misinformation, particularly with respect to age and information sources.

COVID-19 vaccination coverage is generally high among older adults (10, 41). This may partly reflect their greater concern about contracting COVID-19, given the higher risk of severe disease in this age group (42). In addition, earlier access to vaccination and more frequent contact with healthcare facilities, including opportunities to receive advice from physicians, may have promoted vaccine uptake in this population. Interestingly, the present study found that the effect of misinformation on vaccine hesitancy is weak in the younger generation. Among individuals who endorsed misinformation, younger adults in their 20s were more likely to accept vaccination (Figure 2). The result may seem counterintuitive, given the higher vaccination coverage among older adults. Our findings suggest that the younger generation can be more flexible in determining their actions, not being influenced by exposed misinformation. Although Japan did not implement vaccine mandates, easier access to vaccination through universities and workplaces, along with a strong desire to return to ordinary social life, may have attenuated the influence of misinformation on vaccine acceptance in this age group.

Information environments appeared important in both strata of misinformation endorsers and non-endorsers. Although some information sources, such as governments, can reduce vaccine hesitancy only among individuals who did not endorse misinformation, information from medical doctors was associated with higher acceptance regardless of endorsement status, consistent with the role of trusted messengers (43, 44). In contrast, reliance on internet/social media sources was associated with lower acceptance, potentially reflecting heterogeneous information quality, selective exposure, or reinforcement of pre-existing beliefs (45). The association is particularly strong among individuals who endorsed distrust narratives. That may indicate that information channels interact with underlying trust and belief structures (46, 47). At the same time, previous studies showed that quality-assured digital communication can also support vaccine confidence and increase vaccination coverage (48, 49). Furthermore, we found that traditional media, such as newspapers and television, can still exert a strong influence in conveying messages that increase vaccine acceptance, even among individuals who endorse misinformation.

These findings also have implications for digital public health approaches for addressing misinformation. Future public health responses may benefit from digital technologies, including artificial intelligence (AI)-assisted monitoring and communication tools to identify emerging misinformation narratives and support the more timely dissemination of accurate information (50). At the same time, such technologies should be approached cautiously, as AI may also amplify misinformation (51, 52). Their responsible use will require adequate governance, digital literacy, and workforce training. Recent evidence suggests that digital public health training is growing but remains insufficient, underscoring the need for preparation for future infodemics and other public health emergencies (53).

### Limitations

Because this study is cross-sectional, we cannot infer causal direction. Misinformation endorsement may influence vaccination decisions; however, vaccination experiences and social contexts may also shape how individuals perceive and interpret information. Also, pre-existing distrust toward institutions or vaccination may have led some individuals to preferentially seek out, accept, and reinforce misinformation within “echo chambers” (47), rather than misinformation acting as the initial driver of hesitancy. Accordingly, the association observed in this study may reflect bidirectional relationships among distrust, information environments, misinformation endorsement, and vaccination attitudes. In other words, exposure and endorsement of distrustful narratives may function not only as a cause of hesitancy, but also as a consequence or amplifier of pre-existing skepticism.

Moreover, vaccine-related misinformation encompassed a wide range of themes. Yet, the present study focused specifically on distrust-oriented statements about fabrication and concealment, reflecting a combination of acceptance of misinformation, generalized distrust, and critical skepticism toward authoritative institutions (19). The misinformation narratives we asked about in the survey did not include other widely circulated forms of misinformation, such as clinical-biological claims (20, 21). This restricted scope may have led to an underestimation of both the prevalence of misinformation endorsement and the broader vulnerability to vaccine-related misinformation in the study population.

Participants of the survey were recruited from an internet panel and may not fully represent the Japanese population. Despite the weighting approach employed in the present study, selection bias remains possible. Individuals with low digital literacy or limited access to online platforms were less likely to be included. This limitation is particularly relevant for older adults and other socially or medically vulnerable populations, which may have affected the generalizability of the findings. Finally, because vaccination status, attitudes, and other variables were self-reported and were not verified against official vaccination registries, the possibility of misclassification remains. Social desirability bias may also have influenced responses, particularly for vaccination-related items (54, 55).

### Conclusion

Endorsement of vaccine-related misinformation was associated with lower COVID-19 vaccine acceptance in Japan. Furthermore, we found that the majority of misinformation endorsers still accepted vaccination.

Differences in factors associated with vaccine acceptance by endorsement status suggest that public health communication strategies should be tailored to media channels through which information is delivered. In particular, because reliance on internet/social media was more strongly associated with vaccine hesitancy among misinformation endorsers, whereas information from television and newspapers was associated with greater vaccine acceptance in this group, public institutions can strategically use traditional media to disseminate clear and reassuring vaccine messages. Such approaches may be especially important for reaching populations who are susceptible to misinformation and for sustaining vaccine uptake during future public health crises. Public health communication strategies should take into account both information environments and underlying trust dynamics in order to support appropriately informed decision-making.

## Data Availability

The code used for our statistical analyses was available at: https://github.com/yukifuruse1217/jacsis_info. The datasets generated and analyzed for this study are not publicly available due to ethical restrictions, as public sharing would require additional explicit consent from our participants. However, the datasets are available from the corresponding author upon reasonable request.
