# Supplementary material for: Vaccine-related misinformation and attitude toward COVID-19 vaccination in Japan
Source: Front Public Health. 2026 Jul 31;14:1805589. doi: 10.3389/fpubh.2026.1805589 (PMC13473447; doi:10.3389/fpubh.2026.1805589)
Supplement: Supplementary file 1 [file Supplementary_file_1.docx]

**Supplementary Materials**

**Supplementary Table S1 Summary of JACSIS study about vaccine hesitancy and endorsement of vaccine-related misinformation statements**

|  |  |  |  | Vaccination attitude | | | | | Number of endorsed vaccine-related misinformation statements | | | | | | | |
| --- | --- | --- | --- | --- | --- | --- | --- | --- | --- | --- | --- | --- | --- | --- | --- | --- |
| Age group | All participants | Valid responders | Percentage of valid responders | Already vaccinated | Willing but not yet vaccinated | Undecided | Unwilling to get vaccinated | Unable to get vaccinated because of medical reasons | 0 | 1 | 2 | 3 | 4 | 5 | 6 | 7 |
| 15–19 | 645 | 573 | 88.8% | 434 (75.7%) | 37 (6.5%) | 44 (7.7%) | 52 (9.1%) | 6 (1.0%) | 471 (82.2%) | 53 (9.2%) | 24 (4.2%) | 11 (1.9%) | 6 (1.0%) | 2 (0.3%) | 3 (0.5%) | 3 (0.5%) |
| 20–29 | 4,107 | 3,626 | 88.3% | 2,767 (76.3%) | 223 (6.2%) | 307 (8.5%) | 305 (8.4%) | 24 (0.7%) | 3,055 (84.3%) | 286 (7.9%) | 127 (3.5%) | 45 (1.2%) | 31 (0.9%) | 28 (0.8%) | 17 (0.5%) | 37 (1.0%) |
| 30–39 | 4,652 | 4,149 | 89.2% | 3,077 (74.2%) | 270 (6.5%) | 398 (9.6%) | 371 (8.9%) | 33 (0.8%) | 3,589 (86.5%) | 231 (5.6%) | 99 (2.4%) | 61 (1.5%) | 24 (0.6%) | 32 (0.8%) | 39 (0.9%) | 74 (1.8%) |
| 40–49 | 6,078 | 5,454 | 89.7% | 4,330 (79.4%) | 240 (4.4%) | 430 (7.9%) | 388 (7.1%) | 66 (1.2%) | 4,798 (88.0%) | 246 (4.5%) | 115 (2.1%) | 55 (1.0%) | 56 (1.0%) | 46 (0.8%) | 50 (0.9%) | 88 (1.6%) |
| 50–59 | 5,289 | 4,785 | 90.5% | 3,999 (83.6%) | 147 (3.1%) | 291 (6.1%) | 311 (6.5%) | 37 (0.8%) | 4,274 (89.3%) | 207 (4.3%) | 73 (1.5%) | 39 (0.8%) | 28 (0.6%) | 33 (0.7%) | 68 (1.4%) | 63 (1.3%) |
| 60–69 | 5,267 | 4,882 | 92.7% | 4441 (91.0%) | 35 (0.7%) | 160 (3.3%) | 209 (4.3%) | 37 (0.8%) | 4,489 (92.0%) | 177 (3.6%) | 49 (1.0%) | 36 (0.7%) | 19 (0.4%) | 26 (0.5%) | 28 (0.6%) | 58 (1.2%) |
| 70– | 4,962 | 4,706 | 94.8% | 4,387 (93.2%) | 16 (0.3%) | 110 (2.3%) | 164 (3.5%) | 29 (0.6%) | 4,365 (92.8%) | 162 (3.4%) | 46 (1.0%) | 27 (0.6%) | 31 (0.7%) | 23 (0.5%) | 22 (0.5%) | 30 (0.6%) |
| Total | 31,000 | 28,175 | 90.9% | 23,435 (83.2%) | 968 (3.4%) | 1,740 (6.2%) | 1,800 (6.4%) | 232 (0.8%) | 25,041 (88.9%) | 1,362 (4.8%) | 533 (1.9%) | 274 (1.0%) | 195 (0.7%) | 190 (0.7%) | 227 (0.8%) | 353 (1.3%) |

**Supplementary Table S2 Risk factors for endorsement of misinformation statements**

|  |  | Number of endorsed misinformation statements | | | | | | | | Unadjusted | | Adjusted | |
| --- | --- | --- | --- | --- | --- | --- | --- | --- | --- | --- | --- | --- | --- |
| Category | Variable | 0 | 1 | 2 | 3 | 4 | 5 | 6 | 7 | Odds ratio | P-value | Odds ratio | P-value |
| Sex | Male | 12275 (88.5%) | 717 (5.2%) | 272 (2.0%) | 141 (1.0%) | 93 (0.7%) | 83 (0.6%) | 104 (0.7%) | 185 (1.3%) | Reference |  |  |  |
|  | Female | 12766 (89.2%) | 645 (4.5%) | 261 (1.8%) | 133 (0.9%) | 102 (0.7%) | 107 (0.7%) | 123 (0.9%) | 168 (1.2%) | 0.93 (0.87–1.00) | 0.063 | 1.04 (0.95–1.15) | 0.35 |
| Age group | 15-19 | 471 (82.2%) | 53 (9.2%) | 24 (4.2%) | 11 (1.9%) | 6 (1.0%) | 2 (0.3%) | 3 (0.5%) | 3 (0.5%) | **1.51 (1.20–1.88)** | **0.0004** | 1.28 (0.66–2.29) | 0.43 |
|  | 20-29 | 3055 (84.3%) | 286 (7.9%) | 127 (3.5%) | 45 (1.2%) | 31 (0.9%) | 28 (0.8%) | 17 (0.5%) | 37 (1.0%) | **1.32 (1.17–1.49)** | **<0.0001** | 1.11 (0.96–1.29) | 0.17 |
|  | 30–39 | 3589 (86.5%) | 231 (5.6%) | 99 (2.4%) | 61 (1.5%) | 24 (0.6%) | 32 (0.8%) | 39 (0.9%) | 74 (1.8%) | **1.13 (1.00–1.28)** | **0.041** | 1.09 (0.95–1.26) | 0.20 |
|  | 40–49 | 4798 (88.0%) | 246 (4.5%) | 115 (2.1%) | 55 (1.0%) | 56 (1.0%) | 46 (0.8%) | 50 (0.9%) | 88 (1.6%) | Reference |  |  |  |
|  | 50–59 | 4274 (89.3%) | 207 (4.3%) | 73 (1.5%) | 39 (0.8%) | 28 (0.6%) | 33 (0.7%) | 68 (1.4%) | 63 (1.3%) | **0.87 (0.77–0.99)** | **0.030** | 0.90 (0.78–1.04) | 0.16 |
|  | 60–69 | 4489 (92.0%) | 177 (3.6%) | 49 (1.0%) | 36 (0.7%) | 19 (0.4%) | 26 (0.5%) | 28 (0.6%) | 58 (1.2%) | **0.64 (0.56–0.72)** | **<0.0001** | **0.71 (0.60–0.83)** | **<0.0001** |
|  | 70– | 4365 (92.8%) | 162 (3.4%) | 46 (1.0%) | 27 (0.6%) | 31 (0.7%) | 23 (0.5%) | 22 (0.5%) | 30 (0.6%) | **0.56 (0.49–0.65)** | **<0.0001** | **0.67 (0.57–0.80)** | **<0.0001** |
| Educational attainment | Junior high school | 329 (88.7%) | 14 (3.8%) | 12 (3.2%) | 2 (0.5%) | 4 (1.1%) | 5 (1.3%) | 4 (1.1%) | 1 (0.3%) | 1.18 (0.84–1.62) | 0.32 | 1.21 (0.82–1.72) | 0.32 |
|  | High school | 6519 (90.2%) | 300 (4.2%) | 120 (1.7%) | 60 (0.8%) | 48 (0.7%) | 43 (0.6%) | 45 (0.6%) | 92 (1.3%) | Reference |  |  |  |
|  | University/college | 15072 (88.9%) | 811 (4.8%) | 297 (1.8%) | 174 (1.0%) | 110 (0.6%) | 120 (0.7%) | 151 (0.9%) | 218 (1.3%) | **1.15 (1.05–1.26)** | **0.003** | 1.09 (0.98–1.21) | 0.12 |
|  | Postgraduate | 1117 (89.1%) | 62 (4.9%) | 26 (2.1%) | 7 (0.6%) | 9 (0.7%) | 6 (0.5%) | 6 (0.5%) | 20 (1.6%) | 1.12 (0.92–1.35) | 0.27 | 1.05 (0.85–1.30) | 0.63 |
| Income | 0–3 million JPY | 4262 (87.5%) | 262 (5.4%) | 98 (2.0%) | 58 (1.2%) | 30 (0.6%) | 32 (0.7%) | 47 (1.0%) | 81 (1.7%) | **1.21 (1.08–1.35)** | **0.0007** | **1.21 (1.07–1.36)** | **0.002** |
|  | 3–6 million JPY | 7722 (89.4%) | 395 (4.6%) | 153 (1.8%) | 100 (1.2%) | 70 (0.8%) | 49 (0.6%) | 64 (0.7%) | 83 (1.0%) | Reference |  |  |  |
|  | 6–9 million JPY | 4348 (89.0%) | 242 (5.0%) | 92 (1.9%) | 55 (1.1%) | 23 (0.5%) | 33 (0.7%) | 35 (0.7%) | 55 (1.1%) | 1.04 (0.93–1.16) | 0.52 | 0.98 (0.87–1.11) | 0.77 |
|  | 9–12 million JPY | 2015 (88.0%) | 133 (5.8%) | 40 (1.7%) | 15 (0.7%) | 20 (0.9%) | 18 (0.8%) | 20 (0.9%) | 28 (1.2%) | 1.14 (0.99–1.32) | 0.067 | 1.12 (0.96–1.30) | 0.16 |
|  | 12– million JPY | 1268 (87.5%) | 70 (4.8%) | 40 (2.8%) | 9 (0.6%) | 14 (1.0%) | 12 (0.8%) | 15 (1.0%) | 21 (1.4%) | **1.21 (1.02–1.43)** | **0.026** | 1.15 (0.95–1.38) | 0.13 |
| Physical comorbidity | Absent | 10158 (88.5%) | 576 (5.0%) | 196 (1.7%) | 105 (0.9%) | 97 (0.8%) | 76 (0.7%) | 93 (0.8%) | 174 (1.5%) | Reference |  |  |  |
|  | Present | 14883 (89.1%) | 786 (4.7%) | 337 (2.0%) | 169 (1.0%) | 98 (0.6%) | 114 (0.7%) | 134 (0.8%) | 179 (1.1%) | 0.94 (0.87–1.01) | 0.10 | 1.02 (0.93–1.13) | 0.62 |
| Mental illness | Absent | 23398 (89.5%) | 1209 (4.6%) | 441 (1.7%) | 244 (0.9%) | 180 (0.7%) | 161 (0.6%) | 207 (0.8%) | 314 (1.2%) | Reference |  |  |  |
|  | Present | 1643 (81.3%) | 153 (7.6%) | 92 (4.6%) | 30 (1.5%) | 15 (0.7%) | 29 (1.4%) | 20 (1.0%) | 39 (1.9%) | **1.94 (1.72–2.18)** | **<0.0001** | **1.53 (1.31–1.78)** | **<0.0001** |
| COVID-19 | Uninfected | 24678 (89.2%) | 1287 (4.7%) | 489 (1.8%) | 255 (0.9%) | 191 (0.7%) | 190 (0.7%) | 226 (0.8%) | 347 (1.3%) | Reference |  |  |  |
|  | Already infected | 363 (70.9%) | 75 (14.6%) | 44 (8.6%) | 19 (3.7%) | 4 (0.8%) | 0 (0.0%) | 1 (0.2%) | 6 (1.2%) | **3.08 (2.54–3.70)** | **<0.0001** | **2.12 (1.66–2.69)** | **<0.0001** |
| Info. family member | No | 12359 (88.0%) | 698 (5.0%) | 291 (2.1%) | 156 (1.1%) | 105 (0.7%) | 95 (0.7%) | 123 (0.9%) | 212 (1.5%) | Reference |  |  |  |
|  | Yes | 12682 (89.7%) | 664 (4.7%) | 242 (1.7%) | 118 (0.8%) | 90 (0.6%) | 95 (0.7%) | 104 (0.7%) | 141 (1.0%) | **0.84 (0.78–0.90)** | **<0.0001** | 0.96 (0.86–1.07) | 0.43 |
| Info. friend | No | 11783 (88.6%) | 599 (4.5%) | 269 (2.0%) | 126 (0.9%) | 90 (0.7%) | 94 (0.7%) | 126 (0.9%) | 209 (1.6%) | Reference |  |  |  |
|  | Yes | 13258 (89.1%) | 763 (5.1%) | 264 (1.8%) | 148 (1.0%) | 105 (0.7%) | 96 (0.6%) | 101 (0.7%) | 144 (1.0%) | 0.94 (0.87–1.01) | 0.11 | 1.07 (0.96–1.19) | 0.24 |
| Info. medical doctor | No | 17463 (88.4%) | 978 (4.9%) | 382 (1.9%) | 197 (1.0%) | 141 (0.7%) | 135 (0.7%) | 184 (0.9%) | 279 (1.4%) | Reference |  |  |  |
|  | Yes | 7578 (90.0%) | 384 (4.6%) | 151 (1.8%) | 77 (0.9%) | 54 (0.6%) | 55 (0.7%) | 43 (0.5%) | 74 (0.9%) | **0.84 (0.77–0.91)** | **<0.0001** | 0.99 (0.88–1.10) | 0.84 |
| Info. expert | No | 15019 (88.2%) | 824 (4.8%) | 332 (1.9%) | 185 (1.1%) | 139 (0.8%) | 133 (0.8%) | 158 (0.9%) | 240 (1.4%) | Reference |  |  |  |
|  | Yes | 10022 (89.9%) | 538 (4.8%) | 201 (1.8%) | 89 (0.8%) | 56 (0.5%) | 57 (0.5%) | 69 (0.6%) | 113 (1.0%) | **0.83 (0.77–0.89)** | **<0.0001** | 1.00 (0.9–1.12) | 0.94 |
| Info. government | No | 13477 (87.1%) | 836 (5.4%) | 327 (2.1%) | 174 (1.1%) | 126 (0.8%) | 124 (0.8%) | 147 (1.0%) | 256 (1.7%) | Reference |  |  |  |
|  | Yes | 11564 (91.0%) | 526 (4.1%) | 206 (1.6%) | 100 (0.8%) | 69 (0.5%) | 66 (0.5%) | 80 (0.6%) | 97 (0.8%) | **0.67 (0.62–0.72)** | **<0.0001** | **0.70 (0.64–0.78)** | **<0.0001** |
| Info. internet including social media | No | 7382 (89.9%) | 350 (4.3%) | 135 (1.6%) | 66 (0.8%) | 57 (0.7%) | 51 (0.6%) | 53 (0.6%) | 113 (1.4%) | Reference |  |  |  |
|  | Yes | 17659 (88.4%) | 1012 (5.1%) | 398 (2.0%) | 208 (1.0%) | 138 (0.7%) | 139 (0.7%) | 174 (0.9%) | 240 (1.2%) | **1.17 (1.07–1.27)** | **0.0004** | **1.49 (1.33–1.67)** | **<0.0001** |
| Info. newspaper | No | 13918 (87.1%) | 849 (5.3%) | 359 (2.2%) | 180 (1.1%) | 132 (0.8%) | 127 (0.8%) | 161 (1.0%) | 260 (1.6%) | Reference |  |  |  |
|  | Yes | 11123 (91.3%) | 513 (4.2%) | 174 (1.4%) | 94 (0.8%) | 63 (0.5%) | 63 (0.5%) | 66 (0.5%) | 93 (0.8%) | **0.64 (0.59–0.69)** | **<0.0001** | **0.89 (0.80–0.98)** | **0.024** |
| Info. television | No | 4415 (82.9%) | 314 (5.9%) | 167 (3.1%) | 73 (1.4%) | 49 (0.9%) | 61 (1.1%) | 83 (1.6%) | 161 (3.0%) | Reference |  |  |  |
|  | Yes | 20626 (90.3%) | 1048 (4.6%) | 366 (1.6%) | 201 (0.9%) | 146 (0.6%) | 129 (0.6%) | 144 (0.6%) | 192 (0.8%) | **0.51 (0.47–0.56)** | **<0.0001** | **0.53 (0.47–0.59)** | **<0.0001** |
| **Total** |  | **25041 (88.9%)** | **1362 (4.8%)** | **533 (1.9%)** | **274 (1%)** | **195 (0.7%)** | **190 (0.7%)** | **227 (0.8%)** | **353 (1.3%)** |  |  |  |  |

Adjustment was performed for all variables shown in the table. Statistical significance (P < 0.05) is in bold. Info., information source; JPY, Japanese Yen.

**Supplementary Table S3	Risk factors for vaccine hesitancy**

|  |  | Vaccination attitude | | Unadjusted | | Adjusted | |
| --- | --- | --- | --- | --- | --- | --- | --- |
| Category | Variable | Acceptance  (already vaccinated or willing) | Hesitancy  (undecided or unwilling) | Odds ratio | P-value | Odds ratio | P-value |
| Sex | Male | 12042 (87.3%) | 1744 (12.7%) | Reference |  |  |  |
|  | Female | 12361 (87.3%) | 1796 (12.7%) | 1.00 (0.93–1.08) | 0.93 | 1.05 (0.95–1.16) | 0.31 |
| Age group | 15–19 | 471 (83.1%) | 96 (16.9%) | 1.14 (0.90–1.43) | 0.27 | 0.82 (0.38–1.58) | 0.57 |
|  | 20–29 | 2990 (83.0%) | 612 (17.0%) | **1.14 (1.02–1.28)** | **0.022** | 0.97 (0.83–1.14) | 0.74 |
|  | 30–39 | 3347 (81.3%) | 769 (18.7%) | **1.28 (1.15–1.43)** | **<0.0001** | 1.15 (1.00–1.32) | 0.056 |
|  | 40–49 | 4570 (84.8%) | 818 (15.2%) | Reference |  |  |  |
|  | 50–59 | 4146 (87.3%) | 602 (12.7%) | **0.81 (0.72–0.91)** | **<0.0001** | 0.92 (0.79–1.06) | 0.25 |
|  | 60–69 | 4476 (92.4%) | 369 (7.6%) | **0.46 (0.40–0.52)** | **<0.0001** | **0.52 (0.44–0.62)** | **<0.0001** |
|  | 70– | 4403 (94.1%) | 274 (5.9%) | 0.35 (0.30–0.40) | 0.37 | **0.40 (0.33–0.49)** | **<0.0001** |
| Educational attainment | Junior high school | 276 (76.2%) | 86 (23.8%) | **2.07 (1.60–2.65)** | **<0.0001** | **1.93 (1.37–2.67)** | **0.0001** |
|  | High school | 6224 (86.9%) | 938 (13.1%) | Reference |  |  |  |
|  | University/college | 14865 (88.4%) | 1957 (11.6%) | **0.87 (0.80–0.95)** | **0.0015** | **0.82 (0.73–0.91)** | **0.0002** |
|  | Postgraduate | 1127 (90.1%) | 124 (9.9%) | **0.73 (0.60–0.89)** | **0.0018** | **0.78 (0.61–0.98)** | **0.032** |
| Income | 0–3 million JPY | 3977 (83.1%) | 811 (16.9%) | **1.58 (1.43–1.74)** | **<0.0001** | **1.59 (1.41–1.79)** | **<0.0001** |
|  | 3–6 million JPY | 7593 (88.5%) | 982 (11.5%) | Reference |  |  |  |
|  | 6–9 million JPY | 4362 (89.8%) | 494 (10.2%) | **0.88 (0.78–0.98)** | **0.023** | **0.81 (0.72–0.93)** | **0.0018** |
|  | 9–12 million JPY | 2078 (91.3%) | 198 (8.7%) | **0.74 (0.63–0.86)** | **0.0002** | **0.66 (0.55–0.80)** | **<0.0001** |
|  | 12– million JPY | 1300 (90.4%) | 138 (9.6%) | **0.82 (0.68–0.99)** | **0.039** | **0.74 (0.59–0.92)** | **0.0083** |
| Physical comorbidity | Absent | 9643 (84.4%) | 1780 (15.6%) | Reference |  |  |  |
|  | Present | 14760 (89.3%) | 1760 (10.7%) | **0.65 (0.60–0.69)** | **<0.0001** | **0.82 (0.74–0.90)** | **0.0001** |
| Mental illness | Absent | 22758 (87.6%) | 3209 (12.4%) | Reference |  |  |  |
|  | Present | 1645 (83.2%) | 331 (16.8%) | **1.43 (1.26–1.61)** | **<0.0001** | 0.95 (0.79–1.14) | 0.59 |
| COVID-19 | Uninfected | 24004 (87.4%) | 3449 (12.6%) | Reference |  |  |  |
|  | Already infected | 399 (81.4%) | 91 (18.6%) | **1.59 (1.25–1.99)** | **0.0001** | 1.02 (0.73–1.41) | 0.89 |
| Info. family member | No | 11765 (84.5%) | 2164 (15.5%) | Reference |  |  |  |
|  | Yes | 12638 (90.2%) | 1376 (9.8%) | **0.59 (0.55–0.64)** | **<0.0001** | 1.06 (0.95–1.19) | 0.29 |
| Info. friend | No | 11048 (83.8%) | 2134 (16.2%) | Reference |  |  |  |
|  | Yes | 13355 (90.5%) | 1406 (9.5%) | **0.55 (0.51–0.59)** | **<0.0001** | **0.75 (0.67–0.84)** | **<0.0001** |
| Info. medical doctor | No | 16604 (84.6%) | 3011 (15.4%) | Reference |  |  |  |
|  | Yes | 7799 (93.6%) | 529 (6.4%) | **0.37 (0.34–0.41)** | **<0.0001** | **0.57 (0.50–0.65)** | **<0.0001** |
| Info. expert | No | 14320 (84.8%) | 2572 (15.2%) | Reference |  |  |  |
|  | Yes | 10083 (91.2%) | 968 (8.8%) | **0.53 (0.49–0.58)** | **<0.0001** | 0.97 (0.87–1.10) | 0.66 |
| Info. government | No | 12828 (83.6%) | 2521 (16.4%) | Reference |  |  |  |
|  | Yes | 11575 (91.9%) | 1019 (8.1%) | **0.45 (0.41–0.48)** | **<0.0001** | **0.72 (0.65–0.81)** | **<0.0001** |
| Info. internet including social media | No | 6992 (85.7%) | 1165 (14.3%) | Reference |  |  |  |
|  | Yes | 17411 (88.0%) | 2375 (12%) | **0.82 (0.76–0.88)** | **<0.0001** | **1.44 (1.28–1.62)** | **<0.0001** |
| Info. newspaper | No | 13185 (83.2%) | 2657 (16.8%) | Reference |  |  |  |
|  | Yes | 11218 (92.7%) | 883 (7.3%) | **0.39 (0.36–0.42)** | **<0.0001** | **0.75 (0.67–0.84)** | **<0.0001** |
| Info. television | No | 3974 (75.3%) | 1302 (24.7%) | Reference |  |  |  |
|  | Yes | 20429 (90.1%) | 2238 (9.9%) | **0.33 (0.31–0.36)** | **<0.0001** | **0.53 (0.47–0.60)** | **<0.0001** |
| Endorsement of misinformation statements | 0 | 22404 (90.1%) | 2464 (9.9%) | Reference |  |  |  |
|  | 1 | 1068 (79.7%) | 272 (20.3%) | **2.32 (2.01–2.66)** | **<0.0001** | **2.12 (1.77–2.52)** | **<0.0001** |
|  | 2 | 358 (68.5%) | 165 (31.5%) | **4.19 (3.46–5.05)** | **<0.0001** | **3.41 (2.65–4.37)** | **<0.0001** |
|  | 3 | 171 (64.5%) | 94 (35.5%) | **5.00 (3.86–6.43)** | **<0.0001** | **4.47 (3.26–6.08)** | **<0.0001** |
|  | 4 | 114 (59.7%) | 77 (40.3%) | **6.14 (4.57–8.21)** | **<0.0001** | **7.93 (5.48–11.42)** | **<0.0001** |
|  | 5 | 90 (48.1%) | 97 (51.9%) | **9.80 (7.33–13.11)** | **<0.0001** | **10.52 (7.23–15.34)** | **<0.0001** |
|  | 6 | 79 (35.9%) | 141 (64.1%) | **16.23 (12.32–21.53)** | **<0.0001** | **15.79 (11.21–22.42)** | **<0.0001** |
|  | 7 | 119 (34.1%) | 230 (65.9%) | **17.57 (14.06–22.07)** | **<0.0001** | **14.99 (11.34–19.93)** | **<0.0001** |
| **Total** |  | **24403 (87.3%)** | **3540 (12.7%)** |  |  |  |  |

Adjustment was performed for all variables shown in the table. Statistical significance (P < 0.05) is in bold. Info., information source; JPY, Japanese Yen.

**Supplementary Table S4 Risk factors for vaccine hesitancy, separating unwilling and undecided subgroups**

| Category | Variable | Vaccination attitude | | | Association with “Unwilling” vs. “Acceptance” | | | | Association with “Undecided” vs. “Acceptance” | | | |
| --- | --- | --- | --- | --- | --- | --- | --- | --- | --- | --- | --- | --- |
|  |  |  |  |  | Unadjusted | | Adjusted | | Unadjusted | | Adjusted | |
|  |  | Acceptance  (already vaccinated or willing) | Unwilling | Undecided | Odds ratio | P–value | Odds ratio | P–value | Odds ratio | P–value | Odds ratio | P–value |
| Sex | Male | 12042 (87.3%) | 896 (6.5%) | 848 (6.2%) | Reference |  |  |  | Reference |  |  |  |
|  | Female | 12361 (87.3%) | 904 (6.4%) | 892 (6.3%) | 0.98 (0.89–1.08) | 0.72 | 1.13 (0.98–1.29) | 0.088 | 1.02 (0.93–1.13) | 0.62 | 0.99 (0.87–1.12) | 0.87 |
| Age group | 15–19 | 471 (83.1%) | 52 (9.2%) | 44 (7.8%) | 1.30 (0.96–1.76) | 0.091 | 1.11 (0.45–2.74) | 0.81 | 0.99 (0.72–1.37) | 0.97 | 0.58 (0.21–1.62) | 0.30 |
|  | 20–29 | 2990 (83.0%) | 305 (8.5%) | 307 (8.5%) | **1.20 (1.03–1.41)** | **0.022** | 0.94 (0.75–1.19) | 0.62 | 1.09 (0.94–1.27) | 0.27 | 1.01 (0.83–1.23) | 0.94 |
|  | 30–39 | 3347 (81.3%) | 371 (9.0%) | 398 (9.7%) | **1.31 (1.12–1.52)** | **0.0005** | 1.16 (0.94–1.41) | 0.16 | **1.26 (1.09–1.46)** | **0.0014** | 1.14 (0.96–1.36) | 0.13 |
|  | 40–49 | 4570 (84.8%) | 388 (7.2%) | 430 (8.0%) | Reference |  |  |  | Reference |  |  |  |
|  | 50–59 | 4146 (87.3%) | 311 (6.6%) | 291 (6.1%) | 0.88 (0.76–1.03) | 0.12 | 0.99 (0.80–1.22) | 0.91 | **0.75 (0.64–0.87)** | **0.0002** | 0.86 (0.71–1.04) | 0.12 |
|  | 60–69 | 4476 (92.4%) | 209 (4.3%) | 160 (3.3%) | **0.55 (0.46–0.65)** | **<0.0001** | **0.66 (0.52–0.84)** | **0.0008** | **0.38 (0.32–0.46)** | **<0.0001** | **0.42 (0.33–0.52)** | **<0.0001** |
|  | 70– | 4403 (94.1%) | 164 (3.5%) | 110 (2.4%) | **0.44 (0.36–0.53)** | **<0.0001** | **0.59 (0.46–0.77)** | **0.0001** | **0.44 (0.21–0.33)** | **<0.0001** | **0.27 (0.21–0.36)** | **<0.0001** |
| Educational attainment | Junior high school | 276 (76.2%) | 49 (13.5%) | 37 (10.2%) | **2.35 (1.71–3.23)** | **<0.0001** | **1.99 (1.29–3.08)** | **0.002** | **1.78 (1.25–2.54)** | **0.0014** | **1.85 (1.20–2.85)** | **0.0057** |
|  | High school | 6224 (86.9%) | 470 (6.6%) | 468 (6.5%) | Reference |  |  |  | Reference |  |  |  |
|  | University/college | 14865 (88.4%) | 979 (5.8%) | 978 (5.8%) | **0.87 (0.78–0.98)** | **0.019** | **0.80 (0.69–0.93)** | **0.0043** | **0.87 (0.78–0.98)** | **0.022** | **0.82 (0.72–0.94)** | **0.0058** |
|  | Postgraduate | 1127 (90.1%) | 68 (5.4%) | 56 (4.5%) | 0.80 (0.61–1.04) | 0.093 | 0.86 (0.62–1.18) | 0.35 | **0.66 (0.50–0.88)** | **0.0043** | **0.71 (0.52–0.97)** | **0.030** |
| Income | 0–3 million JPY | 3977 (83.1%) | 442 (9.2%) | 369 (7.7%) | **1.83 (1.60–2.10)** | **<0.0001** | **1.69 (1.43–1.99)** | **<0.0001** | **1.35 (1.18–1.55)** | **<0.0001** | **1.52 (1.30–1.78)** | **<0.0001** |
|  | 3–6 million JPY | 7593 (88.5%) | 461 (5.4%) | 521 (6.1%) | Reference |  |  |  | Reference |  |  |  |
|  | 6–9 million JPY | 4362 (89.8%) | 236 (4.9%) | 258 (5.3%) | 0.89 (0.76–1.05) | 0.16 | 0.84 (0.70–1.01) | 0.064 | 0.86 (0.74–1.01) | 0.058 | **0.79 (0.67–0.93)** | **0.0047** |
|  | 9–12 million JPY | 2078 (91.3%) | 90 (4.0%) | 108 (4.7%) | **0.71 (0.57–0.90)** | **0.0042** | **0.65 (0.49–0.85)** | **0.0016** | **0.76 (0.61–0.94)** | **0.011** | **0.67 (0.53–0.85)** | **0.0008** |
|  | 12– million JPY | 1300 (90.4%) | 77 (5.4%) | 61 (4.2%) | 0.98 (0.76–1.25) | 0.85 | 0.92 (0.69–1.24) | 0.60 | **0.68 (0.52–0.90)** | **0.0061** | **0.60 (0.44–0.81)** | **0.0011** |
| Physical comorbidity | Absent | 9643 (84.4%) | 913 (8.0%) | 867 (7.6%) | Reference |  |  |  | Reference |  |  |  |
|  | Present | 14760 (89.3%) | 887 (5.4%) | 873 (5.3%) | **0.63 (0.58–0.70)** | **<0.0001** | **0.80 (0.70–0.92)** | **0.0020** | **0.66 (0.60–0.73)** | **<0.0001** | **0.83 (0.73–0.94)** | **0.0035** |
| Mental illness | Absent | 22758 (87.6%) | 1606 (6.2%) | 1603 (6.2%) | Reference |  |  |  | Reference |  |  |  |
|  | Present | 1645 (83.2%) | 194 (9.8%) | 137 (6.9%) | **1.67 (1.43–1.96)** | **<0.0001** | 1.03 (0.81–1.31) | 0.82 | **1.18 (0.99–1.42)** | **0.070** | 0.89 (0.70–1.13) | 0.33 |
| COVID-19 | Uninfected | 24004 (87.4%) | 1745 (6.4%) | 1704 (6.2%) | Reference |  |  |  | Reference |  |  |  |
|  | Already infected | 399 (81.4%) | 55 (11.2%) | 36 (7.3%) | **1.90 (1.42–2.52)** | **<0.0001** | 1.12 (0.73–1.74) | 0.60 | 1.27 (0.90–1.79) | 0.17 | 0.94 (0.60–1.46) | 0.77 |
| Info. family member | No | 11765 (84.5%) | 1237 (8.9%) | 927 (6.7%) | Reference |  |  |  | Reference |  |  |  |
|  | Yes | 12638 (90.2%) | 563 (4.0%) | 813 (5.8%) | **0.42 (0.38–0.47)** | **<0.0001** | 0.86 (0.73–1.02) | 0.085 | **0.82 (0.74–0.90)** | **<0.0001** | **1.24 (1.07–1.44)** | **0.0036** |
| Info. friend | No | 11048 (83.8%) | 1199 (9.1%) | 935 (7.1%) | Reference |  |  |  | Reference |  |  |  |
|  | Yes | 13355 (90.5%) | 601 (4.1%) | 805 (5.5%) | **0.41 (0.37–0.46)** | **<0.0001** | **0.71 (0.60–0.84)** | **<0.0001** | **0.71 (0.65–0.79)** | **<0.0001** | **0.78 (0.67–0.90)** | **0.0008** |
| Info. medical doctor | No | 16604 (84.6%) | 1560 (8.0%) | 1451 (7.4%) | Reference |  |  |  | Reference |  |  |  |
|  | Yes | 7799 (93.6%) | 240 (2.9%) | 289 (3.5%) | **0.33 (0.29–0.38)** | **<0.0001** | **0.57 (0.47–0.69)** | **<0.0001** | **0.42 (0.37–0.48)** | **<0.0001** | **0.58 (0.49–0.69)** | **<0.0001** |
| Info. expert | No | 14320 (84.8%) | 1365 (8.1%) | 1207 (7.1%) | Reference |  |  |  | Reference |  |  |  |
|  | Yes | 10083 (91.2%) | 435 (3.9%) | 533 (4.8%) | **0.45 (0.41–0.51)** | **<0.0001** | 1.14 (0.96–1.36) | 0.13 | **0.63 (0.56–0.70)** | **<0.0001** | **0.86 (0.74–1.00)** | **0.049** |
| Info. government | No | 12828 (83.6%) | 1390 (9.1%) | 1131 (7.4%) | Reference |  |  |  | Reference |  |  |  |
|  | Yes | 11575 (91.9%) | 410 (3.3%) | 609 (4.8%) | **0.33 (0.29–0.37)** | **<0.0001** | **0.61 (0.51–0.71)** | **<0.0001** | **0.60 (0.54–0.66)** | **<0.0001** | **0.83 (0.72–0.95)** | **0.0068** |
| Info. internet including social media | No | 6992 (85.7%) | 697 (8.5%) | 468 (5.7%) | Reference |  |  |  | Reference |  |  |  |
|  | Yes | 17411 (88.0%) | 1103 (5.6%) | 1272 (6.4%) | **0.64 (0.58–0.70)** | **<0.0001** | **1.38 (1.17–1.62)** | **0.0001** | 1.09 (0.98–1.22) | 0.12 | **1.49 (1.27–1.74)** | **<0.0001** |
| Info. newspaper | No | 13185 (83.2%) | 1406 (8.9%) | 1251 (7.9%) | Reference |  |  |  | Reference |  |  |  |
|  | Yes | 11218 (92.7%) | 394 (3.3%) | 489 (4.0%) | **0.33 (0.29–0.37)** | **<0.0001** | **0.73 (0.62–0.86)** | **0.0002** | **0.46 (0.41–0.51)** | **<0.0001** | **0.77 (0.67–0.89)** | **0.0005** |
| Info. television | No | 3974 (75.3%) | 836 (15.8%) | 466 (8.8%) | Reference |  |  |  | Reference |  |  |  |
|  | Yes | 20429 (90.1%) | 964 (4.3%) | 1274 (5.6%) | **0.22 (0.20–0.25)** | **<0.0001** | **0.38 (0.32–0.45)** | **<0.0001** | **0.53 (0.48–0.59)** | **<0.0001** | **0.75 (0.64–0.88)** | **0.0005** |
| Endorsement of misinformation statements | 0 | 22404 (90.1%) | 1052 (4.2%) | 1412 (5.7%) | Reference |  |  |  | Reference |  |  |  |
|  | 1 | 1068 (79.7%) | 149 (11.1%) | 123 (9.2%) | **2.97 (2.48–3.56)** | **<0.0001** | **2.79 (2.20–3.53)** | **<0.0001** | **1.83 (1.50–2.22)** | **<0.0001** | **1.67 (1.32–2.12)** | **<0.0001** |
|  | 2 | 358 (68.5%) | 111 (21.2%) | 54 (10.3%) | **6.60 (5.29–8.24)** | **<0.0001** | **5.40 (4.00–7.30)** | **<0.0001** | **2.39 (1.79–3.20)** | **<0.0001** | **2.08 (1.44–2.99)** | **0.0001** |
|  | 3 | 171 (64.5%) | 61 (23.0%) | 33 (12.5%) | **7.60 (5.63–10.24)** | **<0.0001** | **7.04 (4.84–10.26)** | **<0.0001** | **3.06 (2.10–4.46)** | **<0.0001** | **2.83 (1.83–4.37)** | **<0.0001** |
|  | 4 | 114 (59.7%) | 51 (26.7%) | 26 (13.6%) | **9.53 (6.81–13.33)** | **<0.0001** | **13.84 (9.09–21.07)** | **<0.0001** | **3.62 (2.36–5.56)** | **<0.0001** | **4.26 (2.53–7.16)** | **<0.0001** |
|  | 5 | 90 (48.1%) | 67 (35.8%) | 30 (16.0%) | **15.85 (11.49–21.88)** | **<0.0001** | **20.40 (13.45–30.95)** | **<0.0001** | **5.29 (3.49–8.02)** | **<0.0001** | **4.57 (2.62–7.95)** | **<0.0001** |
|  | 6 | 79 (35.9%) | 106 (48.2%) | 35 (15.9%) | **28.58 (21.22–38.49)** | **<0.0001** | **30.92 (21.20–45.10)** | **<0.0001** | **7.03 (4.70–10.5)** | **<0.0001** | **6.34 (3.85–10.44)** | **<0.0001** |
|  | 7 | 119 (34.1%) | 203 (58.2%) | 27 (7.7%) | **36.33 (28.73–45.93)** | **<0.0001** | **33.40 (24.73–45.11)** | **<0.0001** | **3.60 (2.36–5.49)** | **<0.0001** | **2.69 (1.56–4.63)** | **0.0004** |
| **Total** |  | **24403 (87.3%)** | **1800 (6.4%)** | **1740 (6.2%)** |  |  |  |  |  |  |  |  |

Adjustment was performed for all variables shown in the table. Statistical significance (P < 0.05) is in bold. Info., information source; JPY, Japanese Yen.

**Supplementary Table S5 Risk factors for vaccine hesitancy, stratified by misinformation endorsement status**

| Category | Variable | Zero misinformation statement endorsement | | | | >1 misinformation statements endorsement | | | |
| --- | --- | --- | --- | --- | --- | --- | --- | --- | --- |
|  |  | Acceptance | Hesitancy | Adjusted odds ratio | P-value | Acceptance | Hesitancy | Adjusted odds ratio | P-value |
| Sex | Male | 10985 (89.9%) | 1232 (10.1%) | Reference |  | 488 (56.6%) | 374 (43.4%) | Reference |  |
|  | Female | 11419 (90.3%) | 1232 (9.7%) | **1.01 (0.91–1.13)** | **<0.0001** | 443 (50.7%) | 430 (49.3%) | 1.30 (1.01–1.67) | 0.91 |
| Age group | 15–19 | 398 (85.0%) | 70 (15.0%) | 0.63 (0.24–1.41) | 0.31 | 34 (69.4%) | 15 (30.6%) | 5.33 (0.64–116.92) | 0.17 |
|  | 20–29 | 2577 (84.8%) | 463 (15.2%) | 1.15 (0.96–1.37) | 0.13 | 183 (64.9%) | 99 (35.1%) | **0.48 (0.31–0.74)** | **0.0009** |
|  | 30–39 | 3018 (84.6%) | 548 (15.4%) | **1.21 (1.04–1.42)** | **0.016** | 159 (49.5%) | 162 (50.5%) | 0.97 (0.68–1.39) | 0.88 |
|  | 40–49 | 4181 (88%) | 571 (12.0%) | Reference |  | 204 (51.5%) | 192 (48.5%) | Reference |  |
|  | 50–59 | 3843 (90.5%) | 403 (9.5%) | 0.88 (0.75–1.05) | 0.15 | 137 (46.1%) | 160 (53.9%) | **1.50 (1.03–2.21)** | **0.037** |
|  | 60–69 | 4222 (94.7%) | 237 (5.3%) | **0.46 (0.38–0.56)** | **<0.0001** | 112 (52.8%) | 100 (47.2%) | 0.87 (0.57–1.34) | 0.54 |
|  | 70– | 4165 (96.0%) | 172 (4.0%) | **0.33 (0.26–0.42)** | **<0.0001** | 102 (57.3%) | 76 (42.7%) | 0.87 (0.54–1.39) | 0.57 |
| Education attainment | Junior high school | 256 (79.0%) | 68 (21%) | **2.13 (1.47–3.04)** | **<0.0001** | 10 (41.7%) | 14 (58.3%) | 1.07 (0.36–3.33) | 0.91 |
|  | High school | 5775 (89.3%) | 692 (10.7%) | Reference |  | 207 (51.9%) | 192 (48.1%) | Reference |  |
|  | University/college | 13662 (91.3%) | 1310 (8.7%) | **0.79 (0.70–0.89)** | **0.0001** | 571 (54.3%) | 481 (45.7%) | 0.84 (0.63–1.12) | 0.22 |
|  | Postgraduate | 1030 (92.3%) | 86 (7.7%) | **0.73 (0.56–0.95)** | **0.020** | 43 (58.9%) | 30 (41.1%) | 0.96 (0.52–1.75) | 0.89 |
| Income | 0–3 million JPY | 3644 (86.8%) | 555 (13.2%) | **1.58 (1.38–1.81)** | **<0.0001** | 142 (42.8%) | 190 (57.2%) | **1.70 (1.22–2.36)** | **0.0016** |
|  | 3–6 million JPY | 6983 (90.9%) | 695 (9.1%) | Reference |  | 287 (56.4%) | 222 (43.6%) | Reference |  |
|  | 6–9 million JPY | 3988 (92.2%) | 338 (7.8%) | **0.79 (0.68–0.92)** | **0.0019** | 178 (61.6%) | 111 (38.4%) | 0.89 (0.64–1.25) | 0.51 |
|  | 9–12 million JPY | 1882 (93.7%) | 126 (6.3%) | **0.63 (0.51–0.78)** | **<0.0001** | 82 (59.9%) | 55 (40.1%) | 0.97 (0.62–1.49) | 0.88 |
|  | 12– million JPY | 1177 (93.4%) | 83 (6.6%) | **0.70 (0.54–0.90)** | **0.0078** | 65 (59.6%) | 44 (40.4%) | 0.93 (0.56–1.51) | 0.76 |
| Physical comorbidity | Absent | 8831 (87.3%) | 1287 (12.7%) | Reference |  | 369 (50.4%) | 363 (49.6%) | Reference |  |
|  | Present | 13573 (92.0%) | 1177 (8.0%) | **0.82 (0.74–0.92)** | **0.0006** | 562 (56.0%) | 441 (44.0%) | **0.74 (0.57–0.95)** | **0.021** |
| Mental illness | Absent | 20992 (90.3%) | 2256 (9.7%) | Reference |  | 812 (53.3%) | 711 (46.7%) | Reference |  |
|  | Present | 1412 (87.2%) | 208 (12.8%) | 0.98 (0.79–1.21) | 0.86 | 119 (56.1%) | 93 (43.9%) | 0.89 (0.58–1.34) | 0.57 |
| COVID-19 | Uninfected | 22106 (90.2%) | 2411 (9.8%) | Reference |  | 884 (53.1%) | 780 (46.9%) | Reference |  |
|  | Already infected | 298 (84.9%) | 53 (15.1%) | 1.30 (0.87–1.89) | 0.19 | 47 (66.2%) | 24 (33.8%) | 0.58 (0.28–1.15) | 0.13 |
| Info. family member | No | 10781 (87.8%) | 1500 (12.2%) | Reference |  | 470 (48.7%) | 495 (51.3%) | Reference |  |
|  | Yes | 11623 (92.3%) | 964 (7.7%) | 1.12 (0.98–1.28) | 0.088 | 461 (59.9%) | 309 (40.1%) | 1.01 (0.75–1.35) | 0.97 |
| Info. friend | No | 10195 (87.1%) | 1506 (12.9%) | Reference |  | 410 (45.7%) | 488 (54.3%) | Reference |  |
|  | Yes | 12209 (92.7%) | 958 (7.3%) | **0.74 (0.65–0.85)** | **<0.0001** | 521 (62.2%) | 316 (37.8%) | 0.75 (0.56–1.02) | 0.065 |
| Info. medical doctor | No | 15238 (87.8%) | 2119 (12.2%) | Reference |  | 628 (48.4%) | 669 (51.6%) | Reference |  |
|  | Yes | 7166 (95.4%) | 345 (4.6%) | **0.56 (0.48–0.65)** | **<0.0001** | 303 (69.2%) | 135 (30.8%) | **0.60 (0.44–0.82)** | **0.0014** |
| Info. expert | No | 13108 (87.9%) | 1811 (12.1%) | Reference |  | 590 (50.6%) | 576 (49.4%) | Reference |  |
|  | Yes | 9296 (93.4%) | 653 (6.6%) | 0.94 (0.82–1.07) | 0.35 | 341 (59.9%) | 228 (40.1%) | 1.02 (0.75–1.39) | 0.91 |
| Info. government | No | 11637 (86.9%) | 1753 (13.1%) | Reference |  | 569 (50.0%) | 568 (50.0%) | Reference |  |
|  | Yes | 10767 (93.8%) | 711 (6.2%) | **0.69 (0.61–0.78)** | **<0.0001** | 362 (60.5%) | 236 (39.5%) | 0.98 (0.73–1.32) | 0.91 |
| Info. internet including social media | No | 6459 (87.9%) | 886 (12.1%) | Reference |  | 263 (56.3%) | 204 (43.7%) | Reference |  |
|  | Yes | 15945 (91.0%) | 1578 (9.0%) | **1.25 (1.10–1.43)** | **0.0009** | 668 (52.7%) | 600 (47.3%) | **2.97 (2.14–4.14)** | **<0.0001** |
| Info. newspaper | No | 11954 (86.6%) | 1857 (13.4%) | Reference |  | 585 (48.9%) | 612 (51.1%) | Reference |  |
|  | Yes | 10450 (94.5%) | 607 (5.5%) | **0.77 (0.67–0.87)** | **0.0001** | 346 (64.3%) | 192 (35.7%) | **0.59 (0.44–0.79)** | **0.0004** |
| Info. television | No | 3526 (80.5%) | 856 (19.5%) | Reference |  | 220 (37.7%) | 364 (62.3%) | Reference |  |
|  | Yes | 18878 (92.2%) | 1608 (7.8%) | **0.56 (0.49–0.65)** | **<0.0001** | 711 (61.8%) | 440 (38.2%) | **0.38 (0.28–0.52)** | **<0.0001** |
| **Total** |  | **22404 (90.1%)** | **2464 (9.9%)** |  |  | **931 (53.7%)** | **804 (46.3%)** |  |  |

Adjustment was performed for all variables shown in the table. Statistical significance (P < 0.05) is in bold. Info., information source; JPY, Japanese Yen.

A

B

**Supplementary Figure S1 Prevalence of vaccine hesitancy and endorsement of vaccine-related misinformation statements in each age group**

A) The prevalence of individuals who endorsed vaccine-related misinformation statements was stratified by vaccination attitude. The percentage of zero misinformation statement endorsement and the total percentage of 1–7 misinformation statements endorsement are labeled. B) The proportions of vaccine acceptance and hesitancy were shown, stratified by the number of endorsed misinformation statements. “1–7” indicates people who endorsed at least one of the seven vaccine-related misinformation statements. The survey results were adjusted for age, residence, educational level, marital status, and self-rated health consciousness to fit the general population in Japan. For the 15–19 age group in panel B, graphs are not shown for endorsement of four or more misinformation statements because the sample size was <10.
